# Fatal Intratumoral Hemorrhage in Mediastinal Diffuse Large B‐Cell Lymphoma During Pregnancy: A Case Report

**DOI:** 10.1111/jog.70232

**Published:** 2026-03-11

**Authors:** Atsuko Yoshida, Takashi Kaji, Tatsuro Sugimoto, Ayuka Mineda, Shiro Fujii, Takeshi Iwasa

**Affiliations:** ^1^ Department of Obstetrics and Gynecology, Institute of Biomedical Sciences Tokushima University Graduate School Tokushima Japan; ^2^ Department of Hematology Endocrinology and Metabolism, Tokushima University Graduate School of Biomedical Sciences Tokushima Japan

**Keywords:** diffuse large B‐cell lymphoma, ECMO, malignant lymphoma, pregnancy, tumoral hemorrhage

## Abstract

We report a pregnant woman with diffuse large B‐cell lymphoma (DLBCL) presenting as a massive mediastinal tumor causing dyspnea. A 34‐year‐old multiparous woman was diagnosed with the tumor at 16 weeks of gestation. During attempts at airway management using ECMO cannulation and intubation, she developed cardiopulmonary arrest due to airway compromise but was resuscitated. R‐CHOP chemotherapy was initiated empirically, and subsequent biopsy confirmed DLBCL, leading to continuation of therapy using DA‐EPOCH‐R. Despite repeated discussions, including the option of pregnancy termination, the patient strongly wished to continue the pregnancy. Partial tumor shrinkage was achieved; however, at 24 weeks, she developed sudden massive hemoptysis and cardiopulmonary arrest, which proved fatal despite resuscitation. Postmortem imaging demonstrated tumoral hemorrhage with bronchial communication. Although tumoral hemorrhage is a rare complication of lymphoma, it should be recognized as a possible cause of sudden death in malignant disease. This case highlightsthe potential for catastrophic tumoral hemorrhage during chemotherapy and the complex ethical dilemmas surrounding pregnancy continuation.

## 1 Introduction

Diffuse large B‐cell lymphoma (DLBCL) is the most common subtype of non‐Hodgkin malignant lymphoma, usually affecting middle‐aged and older adults. A mediastinal presentation tends to occur in younger women, often forming bulky tumors that can cause airway compression or superior vena cava syndrome [1]. Malignant lymphoma during pregnancy is extremely rare (0.8/100000 pregnancies) [1], and management is particularly challenging, as clinicians must balance maternal treatment and fetal safety in the absence of strong evidence [1, 2, 3, 4, 5, 6]. Chemotherapy is considered the standard of care and generally feasible during the second and third trimesters, with minimal fetal risk [2, 4, 5, 6, 7, 8, 9].

Here, we describe a rare and catastrophic case of mediastinal DLBCL in the second trimester of pregnancy. Despite initiation of chemotherapy and multidisciplinary management, the patient died due to fatal tumoral hemorrhage—an exceptionally unusual course not previously reported.

## 2 Case

**FIGURE 1:**
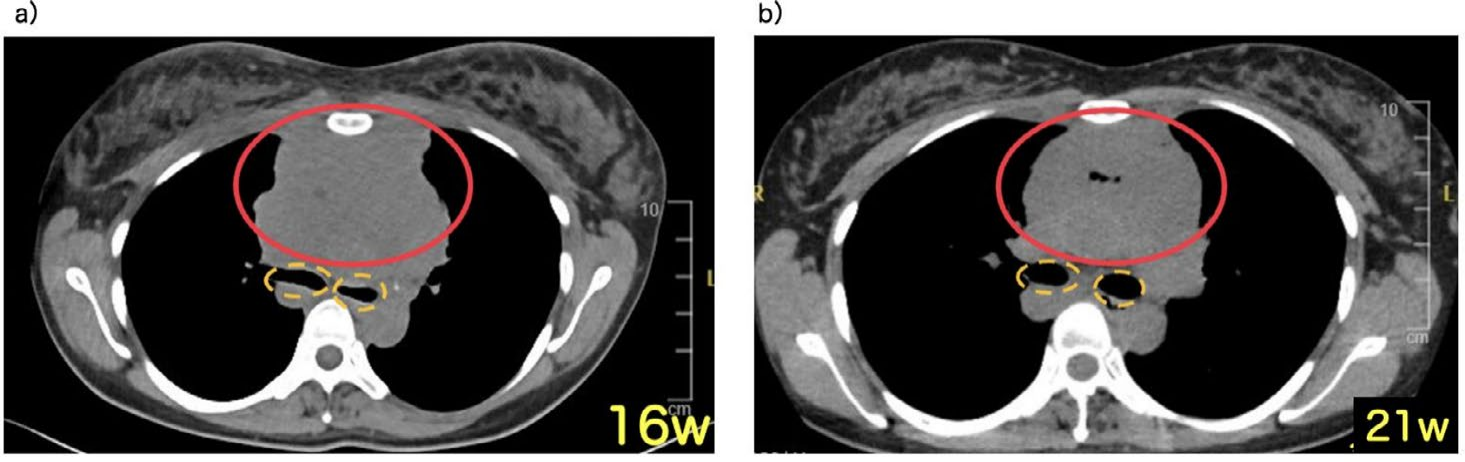
Chest computed tomography. (a) At 16 weeks of gestation (onset), a large anterior mediastinal mass (solid line) and compressed bronchus (dotted line) were observed, accompanied by superior vena cava syndrome and upper body edema. (b) At 21 weeks of gestation (after initiation of chemotherapy), the mediastinal mass (solid line) showed slight reduction in size, and the compressed bronchus (dotted line) was relieved. Upper body edema was also improved. Previously unseen intratumoral air accumulation was observed.

A 34‐year‐old multiparous woman noticed cervical swelling; thyroid function tests were normal at 12 weeks of gestation. At 14 weeks, she developed mild dyspnea and was placed under observation. At 16 weeks, she developed severe nocturnal dyspnea and was transferred to an emergency hospital. Chest computed tomography (CT) revealed a massive mediastinal tumor compressing the airway (Figure 1a). Because she was only able to breathe in the sitting position, she was transferred to our center. Endotracheal intubation was required for airway obstruction; however, due to anticipated difficulty from progressive compression, intubation was attempted under extracorporeal membrane oxygenation (ECMO). During ECMO cannulation, oxygenation could not be maintained, resulting in cardiopulmonary arrest (CPA). Cardiopulmonary resuscitation was immediately initiated, and return of spontaneous circulation was achieved within 7 min following successful intubation and ECMO initiation. Biopsy of the mediastinal tumor was performed at that time. In the intensive care unit (ICU), the patient's husband and mother were counseled regarding her critical condition and the need to consider pregnancy continuation. As malignant lymphoma was suspected and urgent chemotherapy was required for maternal survival, empirical R‐CHOP (rituximab, cyclophosphamide, doxorubicin [hydroxydaunorubicin], vincristine [Oncovin], and prednisone) was initiated. Pathological examination later confirmed the diagnosis of DLBCL. According to the Ann Arbor staging system [9], the disease was considered to be at least stage II bulky disease. Although she remained on ECMO and mechanical ventilation, she regained consciousness and stable vital signs and was able to communicate.

**FIGURE 2:**
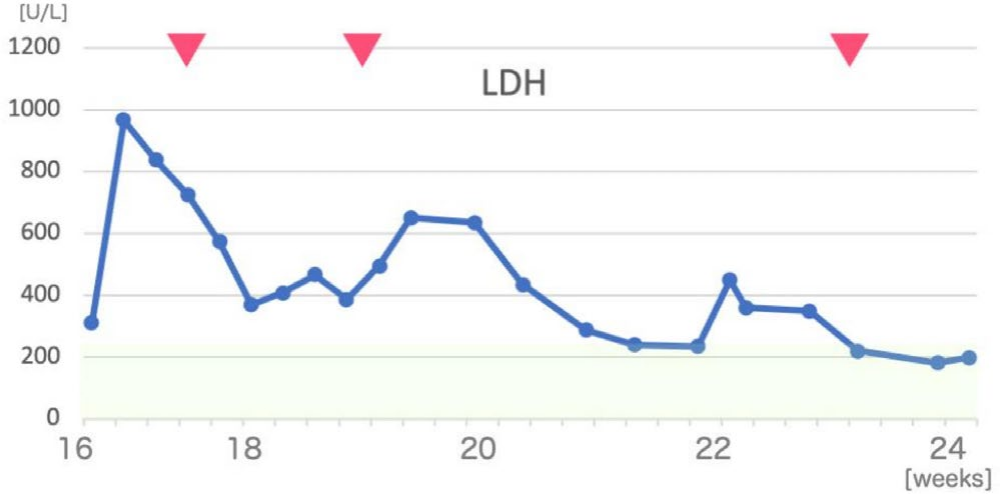
Changes in serum LDH levels during the clinical course. 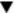: Chemotherapy administered.

Between 16 and 17 weeks, repeated discussions were held with the patient and her family together with the hematology and thoracic surgery teams. The patient consistently expressed a strong wish not to terminate the pregnancy under any circumstances. At 17 weeks, ECMO was successfully withdrawn, and by 18 weeks, partial tumor shrinkage and decreased serum LDH levels were noted, and the patient was successfully extubated (Figure 2). Thereafter, given the limited tumor response to R‐CHOP therapy, the cessation of the decrease in LDH, and the confirmed diagnosis of DLBCL, chemotherapy was switched to DA‐EPOCH‐R (dose‐adjusted etoposide, prednisone, vincristine [Oncovin], cyclophosphamide, doxorubicin [hydroxydaunorubicin], and rituximab). Psychiatric support was also introduced in view of her critical clinical course, prolonged ICU stay, and anxiety regarding the pregnancy.

At 19 weeks, the patient and her husband again received detailed counseling. It was explained that her condition remained critical, continued pregnancy with progressive uterine enlargement would clearly impose an additional burden on respiratory function, the effects of chemotherapy and prior CPA on the fetus were uncertain, and fetal metastasis could not be excluded. The prognosis for both mother and child was highly uncertain. Nonetheless, the patient firmly maintained her decision to continue the pregnancy, and chemotherapy was continued under this policy. At 21 weeks, repeat CT demonstrated further reduction in tumor size, with intratumoral air also visible (Figure 1b). These findings were considered to reflect treatment‐related changes such as tumor shrinkage or cavitation, and continuation of DA‐EPOCH‐R was deemed appropriate. However, the mass remained substantially large, indicating the possibility that the overall therapeutic effect might be limited. Therefore, we remained concerned that, should the disease progress again or treatment resistance become apparent, tumor re‐enlargement could lead to recurrent airway obstruction. Since recurrent airway obstruction could again result in CPA, the patient and her husband were counseled that, in a life‐threatening emergency, perimortem cesarean delivery might need to be considered as part of resuscitative efforts. It was explained that this would represent a second episode of CPA during pregnancy, in which case fetal prognosis would likely be extremely poor, and that maternal resuscitation must be prioritized over fetal considerations. To avoid catastrophic outcomes for both the mother and her fetus, we emphasized the importance of achieving effective treatment for malignant lymphoma and prolonging the gestational period as much as possible. They demonstrated understanding of these explanations.

At 22 weeks of gestation, a second cycle of DA‐EPOCH‐R was administered, and the patient remained hospitalized. At 24 + 2 weeks, she developed sudden massive hemoptysis and CPA. Cardiopulmonary resuscitation was initiated immediately, and return of spontaneous circulation was achieved 4 min later; however, her vital signs remained unstable. A large volume of blood continuously flooded the airway through the endotracheal tube, and tumoral bleeding was judged to be uncontrollable. Preparations for perimortem cesarean delivery were completed 15 min after resuscitation, but massive bleeding and airway obstruction rendered survival unlikely. At that time, the fetus showed persistent severe bradycardia, and fetal survival was also considered unlikely. After being informed of the maternal and fetal conditions, the family declined further invasive interventions, including cesarean delivery, and requested end‐of‐life care. In accordance with their wishes, death was confirmed.

**FIGURE 3:**
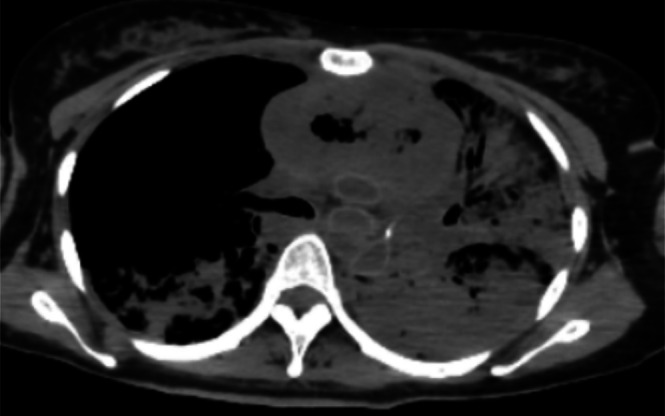
Post‐mortem chest computed tomography. A large amount of air was observed within the mediastinal tumor. Blood had accumulated in the trachea, and the left bronchus was completely obstructed by blood. Findings consistent with aspiration pneumonia were noted, mainly in the left lung field, which showed almost no aeration.

Autopsy imaging demonstrated a reduction in tumor size compared with the time of admission, but intratumoral air and communication with the bronchial tree were evident (Figure 3). The left bronchus was occluded with blood, and aspiration pneumonia–like findings were present in the lungs. The cause of death was determined to be massive tumor hemorrhage and asphyxiation due to the influx of blood into the airway.

## 3 Discussion

DLBCL accounts for approximately 45% of non‐Hodgkin lymphomas [10], and when initial chemotherapy is effective, the prognosis is generally favorable. However, non‐Hodgkin lymphoma during pregnancy is rare, occurring in only 0.8 per 100 000 pregnancies [1]. The standard treatment for lymphoma in pregnancy is systemic chemotherapy [2, 4, 5, 6, 7, 8, 9]. When administered after the period of organogenesis, standard chemotherapy regimens can generally be used during the second and third trimesters. Moreover, many standard chemotherapy regimens can be administered during pregnancy with minimal fetal exposure, and maternal survival outcomes are comparable to those of non‐pregnant patients; consequently, pregnancy itself is not considered to directly affect the prognosis of malignant lymphoma[1,2,4–6]. Similarly, DLBCL generally has a favorable prognosis when an adequate response to chemotherapy is achieved, with a reported 2‐year overall survival of 92%[2,4,5]. However, non‐Hodgkin lymphoma diagnosed during pregnancy is often advanced, partly due to pregnancy‐related diagnostic delay[2,5]. A large multicenter study by Pietro et al. [4] reported a median interval of 40 days from diagnosis to treatment initiation, and Maggen et al. [5] suggested that symptoms such as dyspnea and fatigue may be misattributed to normal physiological changes of pregnancy. In the present case, although treatment was initiated immediately after diagnosis, approximately 4 weeks had elapsed from the onset of cervical swelling to the diagnosis of a mediastinal tumor, during which substantial disease progression was presumed to have occurred, possibly resulting in a missed window for optimal intervention.

Although chemotherapy was initiated and a gradual response was observed, this case is notable for the development of fatal tumor hemorrhage during tumor shrinkage. This outcome may be explained by the occurrence of spontaneous bleeding inherent to malignant neoplasms and chemotherapy‐induced structural weakening of the tumor or involved organs. Such events are extremely rare, and no previous reports have described severe tumor hemorrhage in pregnant women with lymphoma [11, 12]. While airway obstruction and drug toxicity are well‐recognized complications, this case highlights tumor hemorrhage as an additional life‐threatening risk. In addition to massive hemorrhage, the patient experienced asphyxiation, which together resulted in a fatal clinical condition. On CT at 21 weeks of gestation, tumor shrinkage and intratumoral air, interpreted as chemotherapy‐induced tumor necrosis, were taken as signs of treatment response. At the same time, because tumor reduction remained incomplete, we were concerned that tumor re‐enlargement might again cause mechanical airway obstruction. However, in retrospect, the intratumoral air may have represented communication between the tumor and the airway. Ultimately, the mechanism of fatal airway obstruction was not mechanical compression but hemorrhage‐induced asphyxiation. The presence of tumor‐airway communication, combined with uncontrolled tumoral bleeding, led to rapid progression to exsanguination and airway flooding, creating a non‐survivable condition within a brief period of time.

Furthermore, our patient required endotracheal intubation and ECMO from the time of diagnosis, reflecting the severity of her condition. The management dilemma was whether pregnancy continuation itself would affect the maternal prognosis. Previous systematic reviews have suggested that termination of pregnancy does not improve overall survival when chemotherapy is promptly initiated in the second or third trimester [1, 4, 5, 6, 9]. In the present case, treatment was initiated in the same manner as in non‐pregnant patients and subsequently intensified, including regimen modification [9]; therefore, termination of pregnancy would not have affected the treatment for malignant lymphoma. Given the patient's poor initial condition and the risks associated with termination itself, there was no strong evidence to recommend a high‐risk procedure against her wishes. Nevertheless, it remains unresolved whether supporting pregnancy continuation was truly the best decision, given the unpredictable disease course.

In addition, because continuation of pregnancy itself could potentially adversely affect maternal health, we also discussed how pregnancy termination might be managed under ECMO support. Previous reports have described cesarean delivery performed under ECMO support [13, 14]. Given the inability to assume the lithotomy position, the patient's extremely poor general condition, and her history of prior cesarean delivery, planned cesarean delivery was considered as a possible option for pregnancy termination. However, in critically ill pregnant patients, determining the optimal method of pregnancy termination remains ethically and clinically extremely challenging.

Because pregnancy‐associated lymphomas remain rare and heterogeneous, each reported case adds valuable information. The present case highlights the need for further data to clarify the potential prognostic impact of pregnancy and whether earlier delivery or termination should be considered in critically ill patients—even without definitive evidence.

## Author Contributions

Y.A., T.K., S.T., A.M., and T.I., contributed to the perinatal management of this patient. FS contributed to the hematological treatment of this patient. All authors critically revised the report, commented on drafts of the manuscript, and approved the final report.

## Disclosure

The authors have nothing to report.

## Ethics Statement

The authors have nothing to report.

## Consent

Written informed consent for publication of this case report and any accompanying images was obtained from the patient during her lifetime and from her husband. Patient anonymity has been preserved.

## Conflicts of Interest

Takeshi Iwasa is an Editorial Board member of the *Journal of Obstetrics and Gynecology Research* and a co‐author of this article. To minimize bias, Dr. Iwasa was excluded from all editorial decision‐making related to the acceptance of this article for publication.

## Data Availability

The data that support the findings of this study are openly available in Zenodo at https://doi.org/10.5281/zenodo.18335429, reference number 10.5281/zenodo.18335429.
